# Supplementary material for: Mild-moderate CKD is not associated with cognitive impairment in older adults in the Alzheimer’s Disease Neuroimaging Initiative cohort
Source: PLoS One. 2020 Oct 9;15(10):e0239871. doi: 10.1371/journal.pone.0239871 (PMC7546911; doi:10.1371/journal.pone.0239871)
Supplement: S1 File — (DOCX) [file pone.0239871.s001.docx]

**Supplementary data:**

**Supplementary Table 1:** Multivariable linear regression model predicting ADNI-Mem score and ADNI-EF score with participants with and without renal dysfunction.

|  | **Beta estimate for ADNI-Mem score** | **95% confidence interval** | **p value** | **Beta estimate for ADNI-EF score** | **95% confidence interval** | ***p* value** |
| --- | --- | --- | --- | --- | --- | --- |
| Age (+10) | -0.06 | -0.16, 0.03 | 0.20 | -0.25 | -0.36, -0.14 | <0.0001 |
| Female sex | 0.37 | 0.21, 0.52 | <0.0001 | 0.16 | -0.02, 0.33 | 0.08 |
| AA race | 0.23 | 0.11, 0.58 | 0.18 | -0.17 | -0.57, 0.22 | 0.39 |
| Other race | -0.06 | -0.5, 0.37 | 0.77 | 0.01 | -0.49, 0.51 | 0.97 |
| Years of education (+1) | 0.08 | 0.06, 0.10 | <0.0001 | 0.11 | 0.08, 0.13 | <0.0001 |
| BMI (+1) | 0.01 | 0, 0.03 | 0.09 | 0.01 | -0.01,0.03 | 0.23 |
| GFR <60 | 0.06 | -0.1, 0.23 | 0.47 | 0.07 | -0.12, 0.26 | 0.48 |

ADNI-Mem; ADNI composite memory score, ADNI-EF; ADNI composite executive function score, AA; African American, BMI; body mass index (kg/m^2^), eGFR; estimated glomerular filtration rate (ml/min/1.73 m^2^). For race, Caucasian race was the reference group and for eGFR, eGFR ≥60 ml/ min/1.73 m^2^ was the reference group.

**Supplementary Table 2:** Baseline characteristics of participants grouped by cognitive status (with and without cognitive impairment).

| **Participant characteristic** | **All included participants (n = 1181)** | **With cognitive impairment  (n = 805)** | **Without cognitive impairment (n = 376)** | **p-value** |
| --- | --- | --- | --- | --- |
| Age (years) | 73.7 ± 7.1 | 73.3 ± 7.7 | 74.5 ± 5.6 | 0.006 |
| Male sex | 665 (56.3) | 472 (58.6) | 193 (51.3) | 0.018 |
| Ethnicity   Hispanic/Latino   Not Hispanic/Latino   Unknown | 29 (2.5) 1146 (97) 6 (0.5) | 22 (2.7) 779 (96.8) 4 (0.5) | 7 (1.9) 367 (97.6) 2 (0.5) | 0.664 |
| Race  Caucasian  African American  American Indian or Alaskan Native  Asian  Native Hawaiian or Other Pacific Islander  More than one race | 1097 (92.9) 48 (4.1)  3 (0.3) 20 (1.7) 2 (0.2) 11 (0.9) | 751 (93.3) 26 (3.2)  2 (0.2) 15 (1.9) 2 (0.2) 9 (1.1) | 346 (92) 22 (5.9)  1 (0.3) 5 (1.3) 0 (0) 2 (0.5) | 0.244 |
| Right-handed | 1082 (91.6) | 733 (91.1) | 349 (92.8) | 0.308 |
| Marital status   Married  Widowed  Divorced  Never Married  Unknown | 896 (75.9) 150 (12.7) 98 (8.3) 35 (3.0) 2 (0.2) | 638 (79.3) 90 (11.2) 59 (7.3) 17 (2.1) 1 (0.1) | 258 (68.6) 60 (16.0) 39 (10.4) 18 (4.8) 1 (0.3) | 0.001 |
| Years of education | 15.9 ± 2.9 | 15.7 ± 2.9 | 16.3 ± 2.8 | 0.001 |
| BMI (kg/m^2^) | 26.6 ± 4.2 | 26.4 ± 4.3 | 27.0 ± 4.0 | 0.124 |
| FAQ score | 4.7 ± 7.1 | 4.9 ± 7.2 | 4.3 ± 7.0 | 0.153 |
| ADNI-mem score | 0.241 ± 0.874 | -0.115 ± 0.760 | 1.003 ± 0.559 | < 0.001 |
| ADNI-EF score | 0.160 ± 1.026 | -0.110 ± 1.014 | 0.740 ± 0.783 | < 0.001 |
| Serum creatinine (mg/dL) | 1.0 ± 0.2 | 1.0 ± 0.2 | 1.0 ± 0.3 | 0.002 |
| eGFR (CKD EPI) (ml/min/1.73 m^2^) | 75.4 ± 22.9 | 77.2 ± 23.5 | 71.5 ± 21.1 | < 0.001 |
| eGFR (MDRD) (ml/min/1.73 m^2^) | 69.3 ± 17.4 | 70.6 ± 17.5 | 66.3 ± 17.1 | < 0.001 |

ADNI-mem; composite memory score, ADNI-EF; composite executive function score, eGFR; estimated glomerular filtration rate, CKD EPI; Chronic Kidney Disease Epidemiology Collaboration, MDRD; The Modification of Diet in Renal Disease

**Supplementary Table 3:** Distribution of ADNI-mem and ADNI-EF scores by eGFR in ADNI participants

a) with cognitive impairment

|  | All  n = 805 | eGFR<45 n = 42 | eGFR 45-60 n = 169 | eGFR 60-90 n = 398 | eGFR >90 n = 196 | p-value |
| --- | --- | --- | --- | --- | --- | --- |
| ADNI- Mem | -0.115 ± 0.760 | -0.061 ± 0.854 | -0.072 ± 0.846 | -0.124 ± 0.762 | -0.147 ± 0.650 | 0.43 |
| ADNI- EF | -0.110 ± 1.014 | -0.096 ± 1.056 | -0.102 ± 0.950 | -0.160 ± 1.041 | -0.020 ± 1.003 | 0.75 |

b) without cognitive impairment

|  | All  n = 376 | eGFR<45 n = 26 | eGFR 45-60 n = 95 | eGFR 60-90 n = 201 | eGFR >90 n = 54 | p-value |
| --- | --- | --- | --- | --- | --- | --- |
| ADNI- Mem | 1.003 ± 0.559 | 1.117 ± 0.361 | 1.066 ± 0.573 | 0.992 ± 0.586 | 0.883 ± 0.493 | 0.06 |
| ADNI- EF | 0.740 ± 0.783 | 0.778 ± 0.760 | 0.773 ± 0.771 | 0.711 ± 0.810 | 0.771 ± 0.728 | 0.89 |

ADNI-mem; composite memory score, ADNI-EF; composite executive function score, eGFR; estimated glomerular filtration rate (ml/min/1.73 m^2^)

**Supplementary Table 4:** Multivariable linear regression model predicting ADNI-Mem score and ADNI-EF score in participants with and without cognitive impairment.

1. In the group with cognitive impairment
2. With eGFR as a categorical variable. Participants with eGFR <45, 45-60 and >90 are compared to participants with eGFR 61-90 ml/ min/ 1.73 m^2^ taken as the reference group.

|  | **Outcome=ADNI-mem** | | **Outcome=ADNI-EF** | |
| --- | --- | --- | --- | --- |
| **Variable** | **Beta estimate, 95% CI** | **P-value** | **Beta estimate, 95% CI** | **P-value** |

| Age (years) (+10) | -0.11 (-0.20, -0.02) | 0.02 | -0.26 (-0.38, -0.14) | <0.0001 |
| --- | --- | --- | --- | --- |
| Female sex | 0.18 (0.02, 0.35) | 0.03 | 0.09 (-0.13, 0.31) | 0.44 |
| AA race | 0.09 (-0.33, 0.50) | 0.67 | -0.32 (-0.87, 0.24) | 0.27 |
| Other race | 0.22 (-0.20, 0.64) | 0.31 | 0.13 (-0.43, 0.70) | 0.64 |
| Years of education (+1) | 0.05 (0.02, 0.07) | 0.0001 | 0.08 (0.05, 0.11) | <0.0001 |
| BMI (+1) | 0.01 (-0.01, 0.03) | 0.24 | -0.003 (-0.03, 0.02) | 0.77 |
| GFR <45 | -0.07 (-0.43, 0.29) | 0.70 | 0.15 (-0.33, 0.63) | 0.54 |
| GFR 45-60 | 0.05 (-0.15, 0.24) | 0.66 | 0.08 (-0.19, 0.35) | 0.56 |
| GFR >90 | 0.04 (-0.14, 0.23) | 0.64 | 0.25 (0.01, 0.49) | 0.05 |

1. With eGFR as a continuous variable.

|  | **Outcome=ADNI-mem** | | | | **Outcome=ADNI-EF** | | |
| --- | --- | --- | --- | --- | --- | --- | --- |
| **Variable** | **Beta estimate, 95% CI** | | | **P-value** | **Beta estimate, 95% CI** | | **P-value** |
| Age (years) (+10) | | -0.11 (-0.20, -0.02) | 0.01 | | -0.26 (-0.38, -0.14) | <0.0001 | |
| Female sex | | 0.20 (0.04, 0.36) | 0.02 | | 0.13 (-0.09, 0.34) | 0.26 | |
| AA | | 0.09 (-0.33, 0.50) | 0.68 | | -0.28 (-0.83, 0.29) | 0.33 | |
| Other race | | 0.22 (-0.20, 0.64) | 0.31 | | 0.13 (-0.43, 0.70) | 0.65 | |
| Years of education (+1) | | 0.05 (0.02, 0.07) | 0.0001 | | 0.079(0.047,0.111) | <0.0001 | |
| BMI (+1) | | 0.01 (-0.01, 0.03) | 0.24 | | -0.002 (-0.02, 0.02) | 0.89 | |
| eGFR (+10) | | 0.01 (-0.03, 0.04) | 0.68 | | 0.03 (-0.02,0.08) | 0.19 | |

1. In the group without cognitive impairment
2. With eGFR as a categorical variable. Participants with eGFR <45, 45-60 and >90 are compared to participants with eGFR 61-90 ml/ min/ 1.73 m^2^ taken as the reference group.

|  | | | **Outcome=ADNI-mem** | | | **Outcome=ADNI-EF** | | |
| --- | --- | --- | --- | --- | --- | --- | --- | --- |
| **Variable** | | | **Beta estimate, 95% CI** | **P-value** | | **Beta estimate, 95% CI** | **P-value** | |
| Age (years) (+10) | -0.22 (-0.37, -0.08) | | | 0.003 | -0.48 (-0.67, -0.29) | <0.0001 |  |  |
| Female sex | 0.42 (0.26, 0.59) | | | <0.0001 | 0.09 (-0.13, 0.31) | 0.42 |  |  |
| AA race | -0.13 (-0.44, 0.18) | | | 0.41 | -0.50 (-0.91, -0.10) | 0.02 |  |  |
| Other race | -0.37 (-0.97, 0.23) | | | 0.23 | 0.11 (-0.67, 0.90) | 0.78 |  |  |
| Years of education (+1) | 0.07 (0.04, 0.10) | | | <0.0001 | 0.09 (0.05, 0.13) | <0.0001 |  |  |
| BMI (+1) | -0.01 (-0.02, 0.01) | | | 0.58 | 0.02 (-0.01, 0.04) | 0.13 |  |  |
| GFR <45 | -0.11 (-0.47, 0.26) | | | 0.56 | -0.18 (-0.65, 0.29) | 0.46 |  |  |
| GFR 45-60 | -0.02 (-0.20, 0.16) | | | 0.81 | 0.02 (-0.22, 0.26) | 0.86 |  |  |
| GFR >90 | -0.02 (-0.24, 0.20) | | | 0.85 | -0.06 (-0.34, 0.22) | 0.67 |  |  |

1. With eGFR as a continuous variable.

|  | **Outcome=ADNI-mem** | | **Outcome=ADNI-EF** | |
| --- | --- | --- | --- | --- |
| **Variable** | **Beta estimate, 95% CI** | **P-value** | **Beta estimate, 95% CI** | **P-value** |
| Age (years) (+10) | -0.22 (-0.36, -0.07) | 0.004 | -0.48 (-0.66, -0.29) | <0.0001 |
| Female sex | 0.42 (0.26, 0.59) | <0.0001 | 0.09 (-0.13, 0.31) | 0.41 |
| AA | -0.13 (-0.44, 0.18) | 0.41 | -0.5 (-0.9, -0.10) | 0.01 |
| Other race | -0.37 (-0.97, 0.23) | 0.22 | 0.11 (-0.67, 0.89) | 0.78 |
| Years of education (+1) | 0.07 (0.04, 0.10) | <0.0001 | 0.09 (0.05,0.13) | <0.0001 |
| BMI (+1) | -0.01 (-0.02, 0.01) | 0.59 | 0.02 (-0.01, 0.04) | 0.13 |
| eGFR (+10) | 0.002 (-0.04, 0.04) | 0.91 | 0.01 (-0.06, 0.04) | 0.76 |

ADNI-Mem; ADNI composite memory score, ADNI-EF; ADNI composite executive function score, AA; African American, BMI; body mass index (kg/m^2^), eGFR; estimated glomerular filtration rate (ml/min/1.73 m^2^). For race, African American race and other races were compared with Caucasian race.

**Supplementary Table 5:** Correlation analysis between eGFR and ADNI-Mem and ADNI-EF scores in participants with and without cognitive impairment by age, sex, and race.

|  | **With no cognitive impairment** | | **With cognitive impairment** | |
| --- | --- | --- | --- | --- |
| **Subgroups** | **Correlation between eGFR and ADNI-Mem** | **Correlation between eGFR and ADNI-EF** | **Correlation between eGFR and ADNI-Mem** | **Correlation between eGFR and ADNI-EF** |
| **Age <75** | -0.081 (p=0.09) | -0.002 (p=0.95) | -0.129 (p=0.07) | -0.025 (p=0.72) |
| **Age ≥75** | 0.039 (p=0.46) | 0.060 (p=0.25) | -0.092 (p=0.22) | 0.036 (p=0.63) |
| **Male** | 0.026 (p=0.57) | 0.067 (p=0.14) | -0.000 (p=0.99) | 0.007 (p=0.92) |
| **Female** | 0.040 (p=0.46) | 0.004 (p=0.94) | -0.013 (p=0.86) | -0.008 (p=0.91) |
| **Caucasian** | -0.023 (p=0.53) | 0.058 (p=0.11) | -0.103 (p=0.06) | 0.037 (p=0.49) |
| **Non-Caucasian** | -0.091 (p=0.51) | 0.238 (p=0.08) | -0.214 (p=0.26) | -0.155 (p=0.42) |

eGFR; estimated glomerular filtration rate (ml/min/1.73 m^2^).

**Supplementary Table 6**: Multivariable linear regression model with eGFR calculated by MDRD equation in

1. the entire cohort (with cognitive impairment and with no cognitive impairment) with the ADNI-mem and ADNI-EF scores as dependent variables.
2. With eGFR as a categorical variable. Participants with eGFR <45, 45-60 and >90 are compared to participants with eGFR 61-90 ml/ min/ 1.73 m^2^ taken as the reference group.

|  | **Outcome=ADNI-mem** | | **Outcome=ADNI-EF** | |
| --- | --- | --- | --- | --- |
| **Variable** | **Beta estimate, 95% CI** | **P-value** | **Beta estimate, 95% CI** | **P-value** |
| Age (+10) | -0.08 (-0.18, 0.02) | 0.13 | -0.25 (-0.36, -0.13) | <0.0001 |
| Female sex | 0.36 (0.21, 0.51) | <0.0001 | 0.17 (-0.004, 0.35) | 0.06 |
| AA | 0.26 (-0.09, 0.61) | 0.15 | -0.20 (-0.60, 0.21) | 0.34 |
| Other race | -0.07 (-0.51, 0.37) | 0.76 | 0.01 (-0.50, 0.51) | 0.98 |
| Years of education (+1) | 0.08 (0.06, 0.11) | <0.0001 | 0.11 (0.08,0.13) | <0.0001 |
| BMI (+1) | 0.01 (-0.002, 0.03) | 0.09 | 0.01 (-0.01, 0.03) | 0.23 |
| GFR <45 | 0.02 (-0.25, 0.28) | 0.90 | 0.14 (-0.17, 0.45) | 0.37 |
| GFR 45-60 | 0.05 (-0.12, 0.22) | 0.56 | 0.04 (-0.16, 0.24) | 0.71 |
| GFR > 90 | -0.11 (-0.35, 0.12) | 0.35 | 0.07 (-0.21, 0.34) | 0.63 |

ii) With eGFR as a continuous variable.

|  | **Outcome=ADNI-mem** | | **Outcome=ADNI-EF** | | |
| --- | --- | --- | --- | --- | --- |
| **Variable** | **Beta estimate, 95% CI** | **P-value** | **Beta estimate, 95% CI** | **P-value** | |
| Age (+10) | -0.13 (-0.20, -0.05) | 0.001 | -0.29 (-0.39, -0.19) | <0.001 | |
| Female sex | 0.26 (0.14, 0.38) | <0.0001 | 0.10 (-0.06, 0.26) | 0.21 | |
| AA | 0.004 (-0.27, 0.28) | 0.98 | -0.367 (-0.73, -0.004) | 0.05 | |
| Other race | 0.07 (-0.275, 0.41) | 0.70 | 0.12 (-0.34, 0.57) | 0.61 | |
| Years of education (+1) | 0.05 (0.03, 0.07) | <0.0001 | 0.08 (0.06, 0.11) | <0.0001 | |
| BMI (+1) | 0.01 (-0.01, 0.02) | 0.38 | 0.01 (-0.01, 0.02) | 0.53 |  |
| eGFR (+10) | 0.003 (-0.03, 0.04) | 0.87 | 0.02 (-0.03, 0.06) | 0.47 | |

1. the group with cognitive impairment

i) With eGFR as a categorical variable. Participants with eGFR <45, 45-60 and >90 are compared to participants with eGFR 61-90 ml/ min/ 1.73 m^2^ taken as the reference group.

|  | **Outcome=ADNI-mem** | | **Outcome=ADNI-EF** | | |
| --- | --- | --- | --- | --- | --- |
| **Variable** | **Beta estimate, 95% CI** | **P-value** | **Beta estimate, 95% CI** | **P-value** | |
| Age (+10) | -0.12 (-0.21, -0.03) | 0.01 | -0.24 (-0.37, -0.12) | 0.0002 | |
| Female sex | 0.18 (0.02, 0.34) | 0.03 | 0.10 (-0.12, 0.32) | 0.37 | |
| AA | 0.12 (-0.297,0.526) | 0.58 | -0.28 (-0.83, 0.28) | 0.33 | |
| Other race | 0.21 (-0.21, 0.63) | 0.33 | 0.12 (-0.44, 0.69) | 0.67 | |
| Years of education (+1) | 0.05 (0.02, 0.07) | 0.0001 | 0.08 (0.05, 0.11) | <0.0001 | |
| BMI (+1) | 0.01 (-0.01, 0.03) | 0.24 | -0.003 (-0.01, 0.02) | 0.82 |  |
| GFR <45 | -0.06 (-0.36, 0.23) | 0.67 | 0.08 (-0.32, 0.48) | 0.69 | |
| GFR 45-60 | 0.01 (-0.18, 0.19) | 0.93 | -0.03 (-0.28, 0.22) | 0.83 | |
| GFR >90 | -0.06 (-0.29, 0.18) | 0.64 | 0.20 (-0.12, 0.51) | 0.22 | |

1. With eGFR as a continuous variable.

|  | **Outcome=ADNI-mem** | | **Outcome=ADNI-EF** | |
| --- | --- | --- | --- | --- |
| **Variable** | **Beta estimate, 95% CI** | **P-value** | **Beta estimate, 95% CI** | **P-value** |
| Age (+10) | -0.11 (-0.20, -0.02) | 0.02 | -0.25 (-0.38, -0.13) | <0.0001 |
| Female sex | 0.19 (0.03, 0.35) | 0.02 | 0.11 (-0.11, 0.33) | 0.32 |
| AA | 0.09 (-0.32, 0.50) | 0.67 | -0.26 (-0.81, 0.30) | 0.36 |
| Other race | 0.21 (-0.21, 0.63) | 0.32 | 0.13 (-0.44, 0.69) | 0.67 |
| Years of education (+1) | 0.05 (0.02, 0.07) | 0.0001 | 0.08 (0.05, 0.11) | <0.0001 |
| BMI (+1) | 0.01 (-0.01, 0.03) | 0.24 | -0.001 (-0.02,0.02) | 0.91 |
| eGFR (+10) | 0.01 (-0.04, 0.05) | 0.78 | 0.03 (-0.03, 0.09) | 0.36 |

1. the group without cognitive impairment
2. With eGFR as a categorical variable. Participants with eGFR <45, 45-60 and >90 are compared to participants with eGFR 61-90 ml/min/1.73 m^2^ taken as the reference group.

|  | **Outcome=ADNI-mem** | | **Outcome=ADNI-EF** | | |
| --- | --- | --- | --- | --- | --- |
| **Variable** | **Beta estimate, 95% CI** | **P-value** | **Beta estimate, 95% CI** | **P-value** | |
| Age (+10) | -0.23 (-0.38, -0.08) | <0.0001 | -0.50 (-0.70, -0.31) | <0.0001 | |
| Female sex | 0.42 (0.26, 0.58) | <0.0001 | 0.08 (-0.14, 0.29) | 0.48 | |
| AA | -0.10 (-0.42, 0.21) | 0.53 | -0.47 (-0.88, -0.06) | 0.03 | |
| Other race | 0.38 (-0.99, 0.22) | 0.21 | 0.08 (-0.70, 0.87) | 0.83 | |
| Years of education (+1) | 0.07 (0.04, 0.10) | <0.0001 | 0.09 (0.05, 0.13) | <0.0001 | |
| BMI (+1) | -0.01 (-0.02, 0.01) | 0.53 | 0.02 (-0.01, 0.04) | 0.14 |  |
| GFR <45 | -0.03 (-0.29, 0.23) | 0.79 | 0.10 (-0.24, 0.44) | 0.56 | |
| GFR 45-60 | -0.07 (-0.25, 0.10) | 0.42 | 0.001 (-0.23, 0.23) | 0.99 | |
| GFR >90 | -0.12 (-0.41, 0.17) | 0.40 | -0.16 (-0.54, 0.22) | 0.40 | |

1. With eGFR as a continuous variable.

|  | **Outcome=ADNI-mem** | | **Outcome=ADNI-EF** | | |
| --- | --- | --- | --- | --- | --- |
| **Variable** | **Beta Estimate, 95% CI** | **P-value** | **Beta Estimate, 95% CI** | **P-value** | |
| Age (+10) | -0.22 (-0.36, -0.07) | 0.004 | -0.48 (-0.67, -0.29) | <0.0001 | |
| Female sex | 0.42 (0.26, 0.58) | <0.0001 | 0.10 (-0.12, 0.31) | 0.38 | |
| AA | -0.13 (-0.44, 0.18) | 0.41 | -0.50 (-0.90, -0.10) | 0.01 | |
| Other race | -0.37 (-0.97, 0.23) | 0.22 | 0.11 (-0.67, 0.89) | 0.78 | |
| Years of education (+1) | 0.07 (0.04, 0.10) | <0.0001 | 0.09 (0.05, 0.13) | <0.0001 | |
| BMI (+1) | -0.01 (-0.02, 0.01) | 0.59 | 0.02 (-0.01, 0.04) | 0.13 |  |
| eGFR (+10) | -0.002 (-0.05, 0.05) | 0.94 | -0.01 (-0.07, 0.05) | 0.80 | |

ADNI-Mem; ADNI composite memory score, ADNI-EF; ADNI composite executive function score, AA; African American, BMI; body mass index (kg/m^2^), eGFR; estimated glomerular filtration rate (ml/min/1.73 m^2^). For race, African American race and other races were compared with Caucasian race.

**Supplementary Figure 1:** Histogram of eGFR (x-axis) in participants with cognitive impairment (broken line) and without cognitive impairment (solid line) showing identical distribution of eGFR in the two groups.


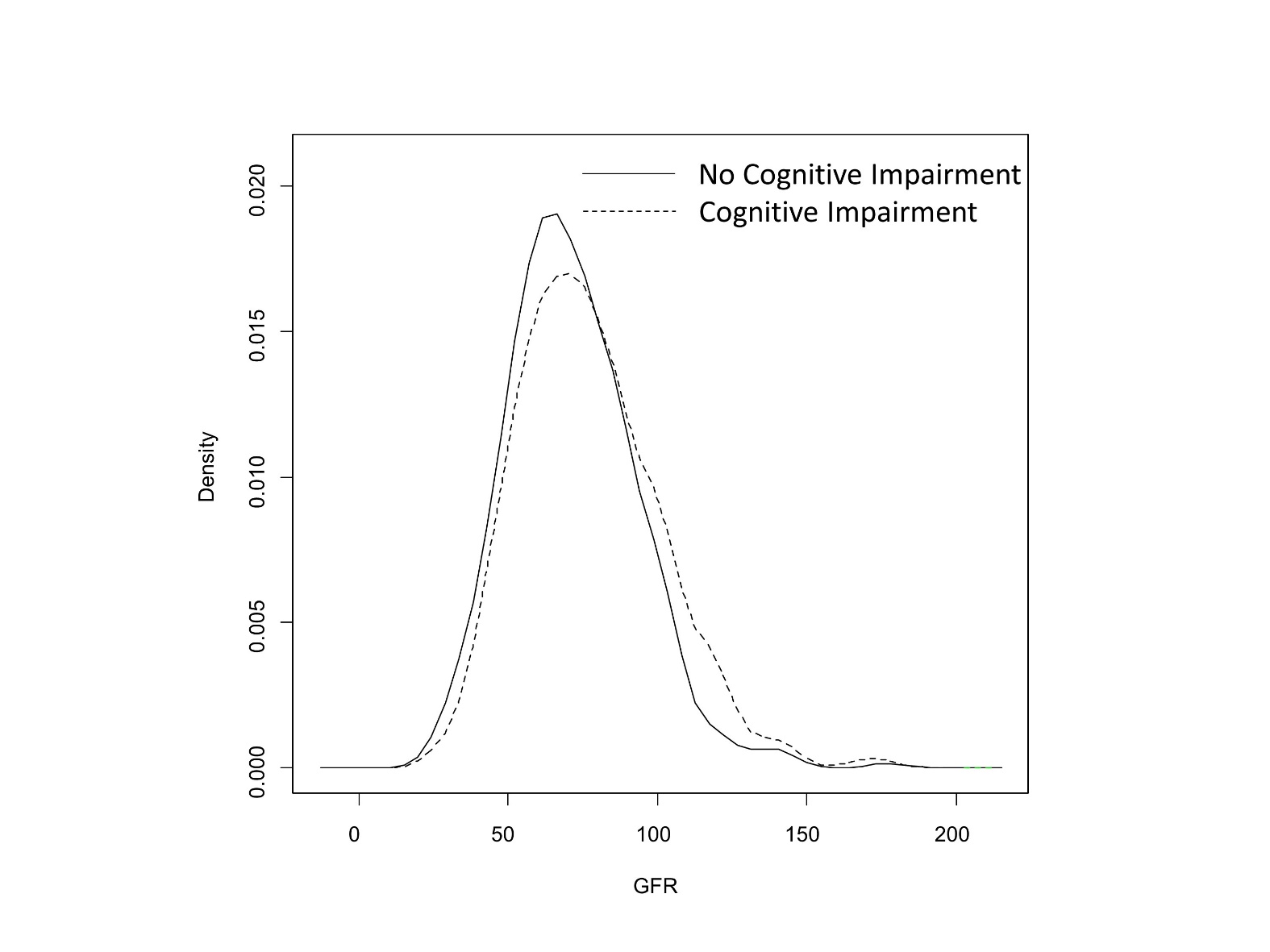


eGFR; estimated glomerular filtration rate (ml/min/1.73 m^2^).

**Supplementary Figure 2:** Scatterplot for ADNI-mem and ADNI-EF scores (y axis) and eGFR (x-axis) in

1. Participants with cognitive impairment.


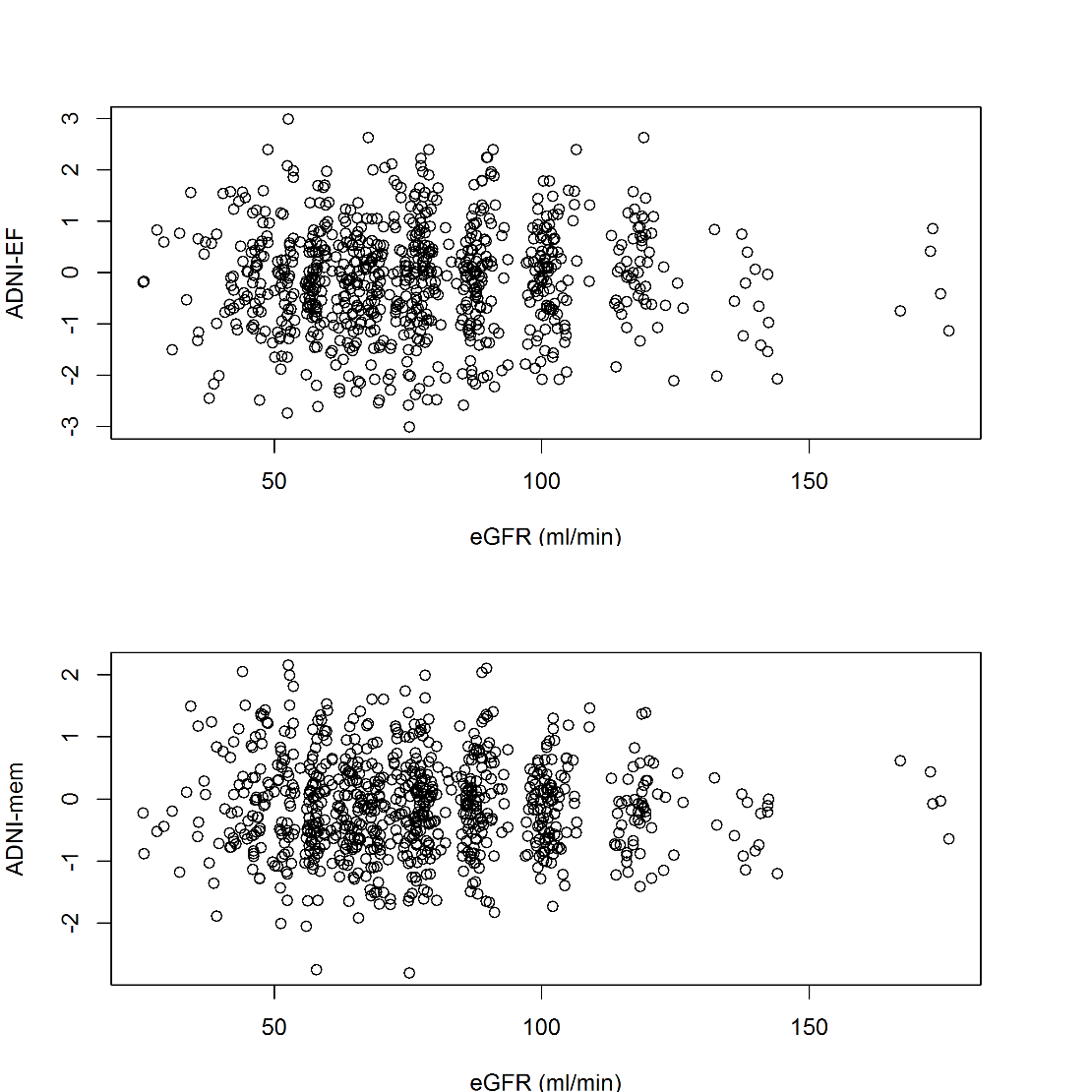


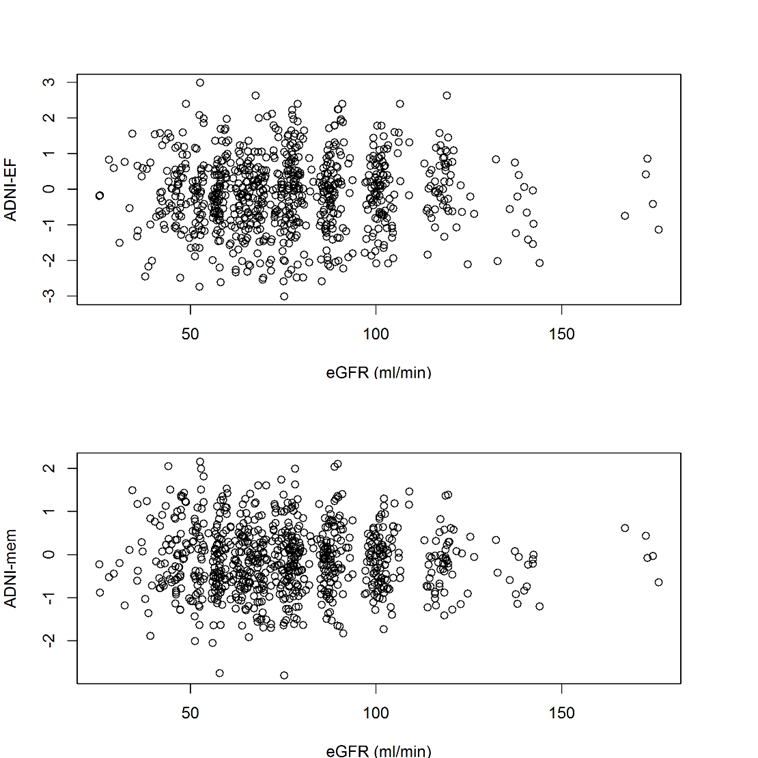


For ADNI-mem, correlation coefficient (r) = 0.025, p= 0.47 and for ADNI-EF r=0.029, p=0.41.

1. Participants without cognitive impairment.


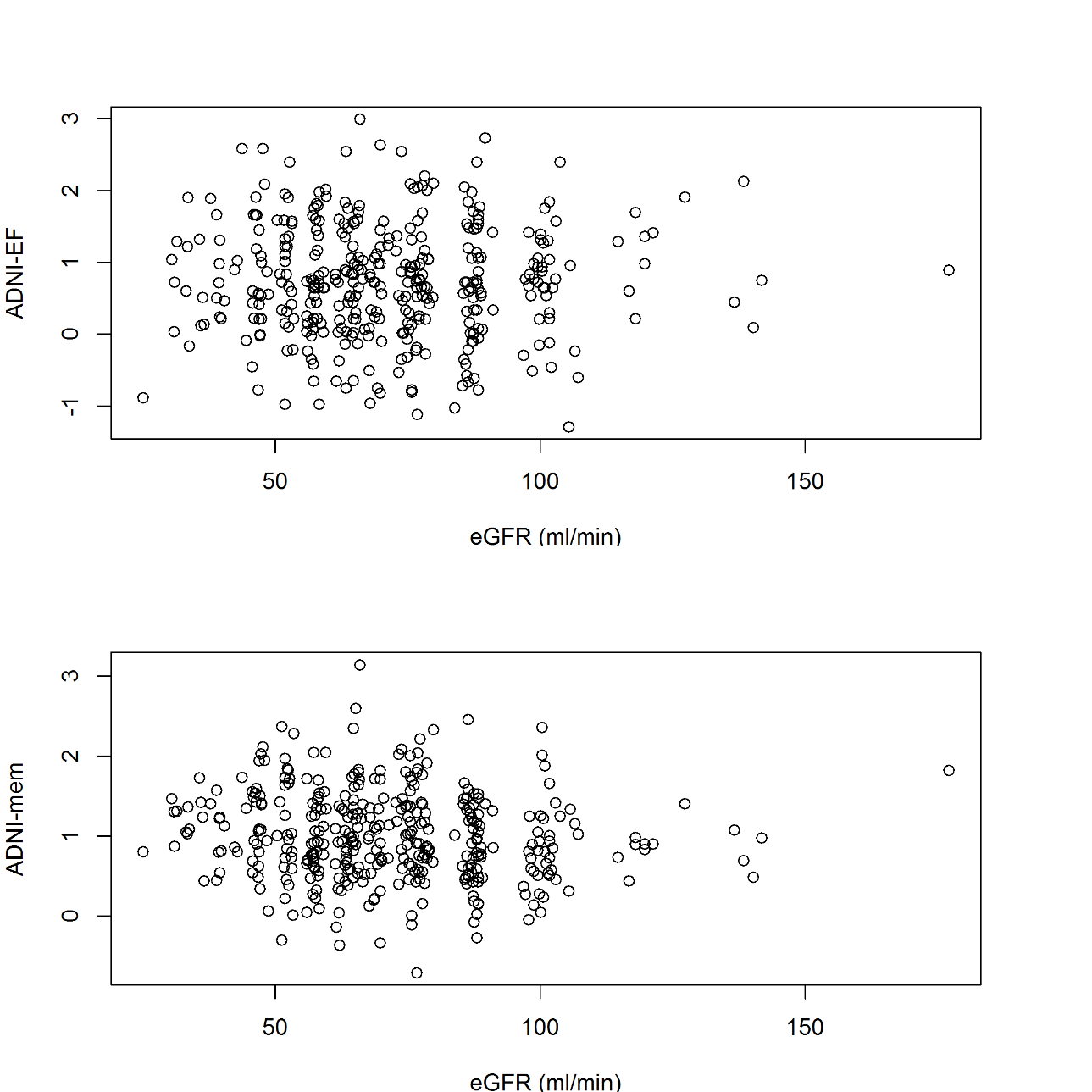


For ADNI-mem r = -0.109 and p=0.03, and for ADNI-EF r = 0.005, p= 0.92

ADNI-mem; composite memory score, ADNI-EF; composite executive function score, eGFR; estimated glomerular filtration rate
