# Supplementary material for: Mild-moderate CKD is not associated with cognitive impairment in older adults in the Alzheimer’s Disease Neuroimaging Initiative cohort
Source: PLoS One. 2020 Oct 9;15(10):e0239871. doi: 10.1371/journal.pone.0239871 (PMC7546911; doi:10.1371/journal.pone.0239871)
Supplement: S2 File — (PDF) [file pone.0239871.s002.pdf]

ADNI Alzheimer's Disease Neuroimaging Initiative

ACKNOWLEDGEMENT LIST FOR ADNI PUBLICATIONS

The Data and Publications Committee, in keeping with the publication policies adopted by the ADNI Steering Committee, here provide lists for standardized acknowledgement. The list consists of three parts: I. ADNI Infrastructure Investigators and Site Investigators, II. DOD ADNI Infrastructure Investigators and Site Investigators and III. ADNI Depression Infrastructure Investigators and Site Investigators. Infrastructure Investigators represent the names responsible for leadership and infrastructure. Site Investigators represent the names of individuals at each recruiting site. All papers, including methodological papers, should have an acknowledgement list that consists of Infrastructure Investigators plus the FULL list.

I. ADNI I, GO, II and III

Part A: Leadership and Infrastructure

Principal Investigator

Michael W. Weiner, MD	UC San Francisco

ATRI PI and Director of Coordinating Center Clinical Core

Paul Aisen, MD	University of Southern California

Executive Committee

Michael Weiner, MD	UC San Francisco
Paul Aisen, MD	University of Southern California
Ronald Petersen, MD, PhD	Mayo Clinic, Rochester
Clifford R. Jack, Jr., MD	Mayo Clinic, Rochester
William Jagust, MD	UC Berkeley
John Q. Trojanowki, MD, PhD	U Pennsylvania
Arthur W. Toga, PhD	USC
Laurel Beckett, PhD	UC Davis
Robert C. Green, MD, MPH	Brigham and Women's Hospital/Harvard Medical School
Andrew J. Saykin, PsyD	Indiana University
John Morris, MD	Washington University St. Louis
Leslie M. Shaw	University of Pennsylvania

ADNI External Advisory Board (ESAB)

Zaven Khachaturian, PhD	Prevent Alzheimer's Disease 2020 (Chair)
Greg Sorensen, MD	Siemens
Maria Carrillo, PhD	Alzheimer's Association
Lew Kuller, MD	University of Pittsburgh
Marc Raichle, MD	Washington University St. Louis
Steven Paul, MD	Cornell University
Peter Davies, MD	Albert Einstein College of Medicine of Yeshiva University
Howard Fillit, MD	AD Drug Discovery Foundation
Franz Hefti, PhD	Acumen Pharmaceuticals
David Holtzman, MD	Washington University St. Louis
M. Marcel Mesulam, MD	Northwestern University
William Potter, MD	National Institute of Mental Health
Peter Snyder, PhD	Brown University

ADNI 3 Private Partner Scientific Board (PPSB)

Veronika Logovinsky, MD, PhD Eli Lilly (Chair)

Data and Publications Committee

Robert C. Green, MD, MPH	BWH/HMS (Chair)

Resource Allocation Review Committee

Tom Montine, MD, PhD	University of Washington (Chair)

Clinical Core Leaders

Ronald Petersen, MD, PhD	Mayo Clinic, Rochester (Core PI)
Paul Aisen, MD	University of Southern California

Clinical Informatics and Operations

Gustavo Jimenez, MBS	USC
Michael Donohue, PhD	USC
Devon Gessert, BS	USC
Kelly Harless, BA	USC
Jennifer Salazar, MBS	USC
Yuliana Cabrera, BS	USC
Sarah Walter, MSc	USC
Lindsey Hergesheimer, BS	USC

Biostatistics Core Leaders and Key Personnel

Laurel Beckett, PhD	UC Davis (Core PI)
Danielle Harvey, PhD	UC Davis
Michael Donohue, PhD	UC San Diego

MRI Core Leaders and Key Personnel

Clifford R. Jack, Jr., MD	Mayo Clinic, Rochester (Core PI)
Matthew Bernstein, PhD	Mayo Clinic, Rochester
Nick Fox, MD	University of London
Paul Thompson, PhD	UCLA School of Medicine
Norbert Schuff, PhD	UCSF MRI
Charles DeCArli, MD	UC Davis
Bret Borowski, RT	Mayo Clinic
Jeff Gunter, PhD	Mayo Clinic
Matt Senjem, MS	Mayo Clinic
Prashanthi Vemuri, PhD	Mayo Clinic
David Jones, MD	Mayo Clinic
Kejal Kantarci	Mayo Clinic
Chad Ward	Mayo Clinic

PET Core Leaders and Key Personnel

William Jagust, MD	UC Berkeley (Core PI)
Robert A. Koeppe, PhD	University of Michigan
Norm Foster, MD	University of Utah
Eric M. Reiman, MD	Banner Alzheimer's Institute
Kewei Chen, PhD	Banner Alzheimer's Institute
Chet Mathis, MD	University of Pittsburgh

Susan Landau, PhD UC Berkeley

Neuropathology Core Leaders

John C. Morris, MD	Washington University St. Louis
Nigel J. Cairns, PhD, FRCPath	Washington University St. Louis
Erin Franklin, MS, CCRP	Washington University St. Louis
Lisa Taylor-Reinwald, BA, HTL (ASCP) – Past Investigator	Washington University St. Louis

Biomarkers Core Leaders and Key Personnel

Leslie M. Shaw, PhD	UPenn School of Medicine
John Q. Trojanowki, MD, PhD	UPenn School of Medicine
Virginia Lee, PhD, MBA	UPenn School of Medicine
Magdalena Korecka, PhD	UPenn School of Medicine
Michal Figurski, PhD	UPenn School of Medicine

Informatics Core Leaders and Key Personnel

Arthur W. Toga, PhD	USC (Core PI)
Karen Crawford	USC
Scott Neu, PhD	USC

Genetics Core Leaders and Key Personnel

Andrew J. Saykin, PsyD	Indiana University
Tatiana M. Foroud, PhD	Indiana University
Steven Potkin, MD UC	UC Irvine
Li Shen, PhD	Indiana University
Kelley Faber, MS, CCRC	Indiana University
Sungeun Kim, PhD	Indiana University
Kwangsik Nho, PhD	Indiana University

Initial Concept Planning & Development

Michael W. Weiner, MD	UC San Francisco
Lean Thal, MD	UC San Diego
Zaven Khachaturian, PhD	Prevent Alzheimer's Disease 2020

Early Project Proposal Development

Leon Thal, MD	UC San Diego
Neil Buckholtz	National Institute on Aging
Michael W. Weiner, MD	UC San Francisco
Peter J. Snyder, PhD	Brown University
William Potter, MD	National Institute of Mental Health
Steven Paul, MD	Cornell University
Marilyn Albert, PhD	Johns Hopkins University
Richard Frank, MD, PhD	Richard Frank Consulting
Zaven Khachaturian, PhD	Prevent Alzheimer's Disease 2020

NIA

John Hsiao, MD	National Institute on Aging

Part B: Investigators By Site

Oregon Health & Science University:
Joseph Quinn, MD
Lisa C. Silbert, MD
Betty Lind, BS
Jeffrey A. Kaye, MD, A. – Past Investigator
Raina Carter, BA – Past Investigator
Sara Dolen, BS – Past Investigator

University of Southern California:
Lon S. Schneider, MD
Sonia Pawluczyk, MD
Mauricio Becerra, BS
Liberty Teodoro, RN
Bryan M. Spann, DO, PhD – Past Investigator

University of California – San Diego:
James Brewer, MD, PhD
Helen Vanderswag, RN
Adam Fleisher, MD – Past Investigator

University of Michigan:
Jaimie Ziolkowski, MA, BS, TLLP
Judith L. Heidebrink, MD, MS
Joanne L. Lord, LPN, BA, CCRC – Past Investigator

Mayo Clinic, Rochester:
Ronald Petersen, MD, PhD
Sara S. Mason, RN
Colleen S. Albers, RN
David Knopman, MD
Kris Johnson, RN – Past Investigator

Baylor College of Medicine:
Javier Villanueva-Meyer, MD
Valory Pavlik, PhD
Nathaniel Pacini, MA
Ashley Lamb, MA
Joseph S. Kass, MD, LD, FAAN
Rachelle S. Doody, MD, PhD – Past Investigator
Victoria Shibley, MS – Past Investigator
Munir Chowdhury, MBBS, MS – Past Investigator
Susan Rountree, MD – Past Investigator
Mimi Dang, MD – Past Investigator

Columbia University Medical Center:
Yaakov Stern, PhD
Lawrence S. Honig, MD, PhD
Karen L. Bell, MD
Randy Yeh, MD

Washington University, St. Louis:
Beau Ances, MD, PhD, MSc
John C. Morris, MD
David Winkfield, BS
Maria Carroll, RN, MSN, GCNS-BC
Angela Oliver, RN, BSN, MSG
Mary L. Creech, RN, MSW – Past Investigator
Mark A. Mintun, MD – Past Investigator
Stacy Schneider, APRN, BC, GNP – Past Investigator

University of Alabama - Birmingham:
Daniel Marson, JD, PhD
David Geldmacher, MD
Marissa Natelson Love, MD
Randall Griffith, PhD, ABPP – Past Investigator
David Clark, MD – Past Investigator
John Brockington, MD – Past Investigator

Mount Sinai School of Medicine:
Hillel Grossman, MD
Effie Mitsis, PhD – Past Investigator

Rush University Medical Center:
Raj C. Shah, MD
Melissa Lamar, PhD
Patricia Samuels

Wien Center:
Ranjan Duara, MD
Maria T. Greig-Custo, MD
Rosemarie Rodriguez, PhD

Johns Hopkins University:
Marilyn Albert, PhD
Chiadi Onyike, MD
Daniel D'Agostino II, BS
Stephanie Kielb, BS – Past Investigator

New York University:
Martin Sadowski, MD, PhD
Mohammed O. Sheikh, MD
Jamika Singleton-Garvin, CCRP
Anaztasia Ulysse
Mrunalini Gaikwad

Duke University Medical Center:
P. Murali Doraiswamy, MBBS, FRCP
Jeffrey R. Petrella, MD
Olga James, MD
Salvador Borges-Neto, MD
Terence Z. Wong, MD – Past Investigator
Edward Coleman – Past Investigator

University of Pennsylvania:
Jason H. Karlawish, MD
David A. Wolk, MD
Sanjeev Vaishnavi, MD
Christopher M. Clark, MD – Past Investigator
Steven E. Arnold, MD – Past Investigator

University of Kentucky:
Charles D. Smith, MD
Greg Jicha, MD
Peter Hardy, PhD
Riham El Khouli, MD
Elizabeth Oates, MD
Gary Conrad, MD

University of Pittsburgh:
Oscar L. Lopez, MD
MaryAnn Oakley, MA
Donna M. Simpson, CRNP, MPH

University of Rochester Medical Center:
Anton P. Porsteinsson, MD
Kim Martin, RN
Nancy Kowalksi, MS, RNC
Melanie Keltz, RN
Bonnie S. Goldstein, MS, NP – Past Investigator
Kelly M. Makino, BS – Past Investigator
M. Saleem Ismail, MD – Past Investigator
Connie Brand, RN – Past Investigator

University of California Irvine IMIND:
Gaby Thai, MD
Aimee Pierce, MD
Beatriz Yanez, RN
Elizabeth Sosa, PhD
Megan Witbracht, PhD

University of Texas Southwestern Medical School:
Kyle Womack, MD
Dana Mathews, MD, PhD
Mary Quiceno, MD

Emory University:
Allan I. Levey, MD, PhD
James J. Lah, MD, PhD
Janet S. Cellar, DNP, PMHCNS-BC

University of Kansas, Medical Center:
Jeffrey M. Burns, MD
Russell H. Swerdlow, MD
William M. Brooks, PhD

University of California, Los Angeles:
Ellen Woo, PhD
Daniel H.S. Silverman, MD, PhD
Edmond Teng, MD, PhD
Sarah Kremen, MD
Liana Apostolova, MD – Past Investigator
Kathleen Tingus, PhD – Past Investigator
Po H. Lu, PsyD – Past Investigator
George Bartzokis, MD – Past Investigator

Mayo Clinic, Jacksonville:
Neill R Graff-Radford, MBBCH, FRCP (London)
Francine Parfitt, MSH, CCRC
Kim Poki-Walker, BA

Indiana University:
Martin R. Farlow, MD
Ann Marie Hake, MD
Brandy R. Matthews, MD – Past Investigator
Jared R. Brosch, MD
Scott Herring, RN, CCRC

Yale University School of Medicine:
Christopher H. van Dyck, MD
Richard E. Carson, PhD
Pradeep Varma, MD

McGill Univ., Montreal-Jewish General Hospital:
Howard Chertkow, MD
Howard Bergman, MD
Chris Hosein, MEd

Sunnybrook Health Sciences, Ontario:
Sandra Black, MD, FRCPC
Bojana Stefanovic, PhD
Chris (Chinthaka) Heyn, BSC, PhD, MD, FRCPC

U.B.C. Clinic for AD & Related Disorders:
Ging-Yuek Robin Hsiung, MD, MHSc, FRCPC
Benita Mudge, BS
Vesna Sossi, PhD
Howard Feldman, MD, FRCPC – Past Investigator
Michele Assaly, MA – Past Investigator

Cognitive Neurology - St. Joseph's, Ontario:
Elizabeth Finger, MD
Stephen Pasternack, MD, PhD
William Pavlosky, MD
Irina Rachinsky, MD – Past Investigator
Dick Drost, PhD – Past Investigator
Andrew Kertesz, MD – Past Investigator

Cleveland Clinic Lou Ruvo Center for Brain Health:
Charles Bernick, MD, MPH
Donna Munic, PhD

Northwestern University:
Marek-Marsel Mesulam, MD
Emily Rogalski, PhD
Kristine Lipowski, MA
Sandra Weintraub, PhD
Borna Bonakdarpour, MD
Diana Kerwin, MD – Past Investigator
Chuang-Kuo Wu, MD, PhD – Past Investigator
Nancy Johnson, PhD – Past Investigator

Premiere Research Inst (Palm Beach Neurology):
Carl Sadowsky, MD
Teresa Villena, MD

Georgetown University Medical Center:
Raymond Scott Turner, MD, PhD
Kathleen Johnson, NP
Brigid Reynolds, NP

Brigham and Women's Hospital:
Reisa A. Sperling, MD
Keith A. Johnson, MD
Gad A. Marshall, MD

Stanford University:
Jerome Yesavage, MD
Joy L. Taylor, PhD
Steven Chao, MD, PhD
Barton Lane, MD – Past Investigator
Allyson Rosen, PhD – Past Investigator
Jared Tinklenberg, MD – Past Investigator

Banner Sun Health Research Institute:
Edward Zamrini, MD
Christine M. Belden, PsyD
Sherye A. Sirrel, CCRC

Boston University:
Neil Kowall, MD
Ronald Killiany, PhD
Andrew E. Budson, MD
Alexander Norbash, MD – Past Investigator
Patricia Lynn Johnson, BA – Past Investigator

Howard University:
Thomas O. Obisesan, MD, MPH
Ntekim E. Oyonumo, MD, PhD
Joanne Allard, PhD
Olu Ogunlana, BPharm

Case Western Reserve University:
Alan Lerner, MD
Paula Ogrocki, PhD
Curtis Tatsuoka, PhD
Parianne Fatica, BA, CCRC

University of California, Davis – Sacramento:
Evan Fletcher, PhD
Pauline Maillard, PhD
John Olichney, MD
Charles DeCarli, MD
Owen Carmichael, PhD – Past Investigator

Neurological Care of CNY:
Smita Kittur, MD – Past Investigator

Parkwood Institute:
Michael Borrie, MB ChB
T-Y Lee, PhD
Dr Rob Bartha, PhD

University of Wisconsin:
Sterling Johnson, PhD
Sanjay Asthana, MD

Cynthia M. Carlsson, MD, MS

Banner Alzheimer's Institute:
Pierre Tariot, MD
Anna Burke, MD
Joel Hetelle, BS
Kathryn DeMarco, BS
Nadira Trncic, MD, PhD, CCRC – Past Investigator
Adam Fleisher, MD – Past Investigator
Stephanie Reeder, BA – Past Investigator

Dent Neurologic Institute:
Vernice Bates, MD
Horacio Capote, MD
Michelle Rainka, PharmD, CCRP

Ohio State University:
Douglas W. Scharre, MD
Maria Kataki, MD, PhD
Rawan Tarawneh, MD

Albany Medical College:
Earl A. Zimmerman, MD
Dzintra Celmins, MD
David Hart, MD

Hartford Hospital, Olin Neuropsychiatry Research Center:
Godfrey D. Pearlson, MD
Karen Blank, MD
Karen Anderson, RN

Dartmouth-Hitchcock Medical Center:
Laura A. Flashman, PhD
Marc Seltzer, MD
Mary L. Hynes, RN, MPH
Robert B. Santulli, MD – Past Investigator

Wake Forest University Health Sciences:
Kaycee M. Sink, MD, MAS
Mia Yang, MD
Akiva Mintz, MD, PhD

Rhode Island Hospital:
Brian R. Ott, MD
Geoffrey Tremont, PhD
Lori A. Daiello, Pharm.D, ScM

Butler Hospital:
Courtney Bodge, PhD
Stephen Salloway, MD, MS
Paul Malloy, PhD
Stephen Correia, PhD
Athena Lee, PhD

UC San Francisco:
Howard J. Rosen, MD
Bruce L. Miller, MD
David Perry, MD

Medical University South Carolina:
Jacobo Mintzer, MD, MBA
Kenneth Spicer, MD, PhD
David Bachman, MD

St. Joseph's Health Care:
Elizabeth Finger, MD
Stephen Pasternak, MD
Irina Rachinsky, MD
John Rogers, MD
Andrew Kertesz, MD – Past Investigator
Dick Drost, MD – Past Investigator

Nathan Kline Institute
Nunzio Pomara, MD
Raymundo Hernando, MD
Antero Sarrael, MD

University of Iowa College of Medicine
Delwyn D. Miller, PharmD, MD
Karen Ekstam Smith, RN
Hristina Koleva, MD
Ki Won Nam, MD
Hyungsub Shim, MD
Susan K. Schultz, MD – Past Investigator

Cornell University
Norman Relkin, MD, PhD
Gloria Chiang, MD
Michael Lin, MD
Lisa Ravdin, PhD

University of South Florida: USF Health Byrd Alzheimer's Institute
Amanda Smith, MD
Christi Leach, MD
Balebail Ashok Raj, MD – Past Investigator
Kristin Fargher, MD – Past Investigator

DOD ADNI

Part A: Leadership and Infrastructure

Principal Investigator

Michael W. Weiner, MD	University of California, San Francisco

ATRI PI and Director of Coordinating Center Clinical Core

Paul Aisen, MD	University of Southern California

Executive Committee

Michael Weiner, MD	UC San Francisco
Paul Aisen, MD	University of Southern California
Ronald Petersen, MD, PhD	Mayo Clinic, Rochester
Robert C. Green, MD, MPH	Brigham and Women's Hospital/ Harvard Medical School
Danielle Harvey, PhD	UC Davis
Clifford R. Jack, Jr., MD	Mayo Clinic, Rochester
William Jagust, MD	UC Berkeley
John C. Morris, MD	Washington University St. Louis
Andrew J. Saykin, PsyD	Indiana University
Leslie M. Shaw, PhD	Perelman School of Medicine, UPenn
Arthur W. Toga, PhD	USC
John Q. Trojanowki, MD, PhD	Perelman School of Medicine, University of Pennsylvania

Psychological Evaluation/PTSD Core

Thomas Neylan, MD	UC San Francisco

Traumatic Brain Injury/TBI Core

Jordan Grafman, PhD	Rehabilitation Institute of Chicago, Feinberg School of Medicine, Northwestern University

Data and Publication Committee (DPC)

Robert C. Green, MD, MPH	BWH/HMS (Chair)

Resource Allocation Review Committee

Tom Montine, MD, PhD	University of Washington (Chair)

Clinical Core Leaders

Michael Weiner MD	Core PI
Ronald Petersen, MD, PhD	Mayo Clinic, Rochester (Core PI)
Paul Aisen, MD	University of Southern California

Clinical Informatics and Operations

Gustavo Jimenez, MBS	USC
Michael Donohue, PhD	USC
Devon Gessert, BS	USC

Kelly Harless, BA	USC
Jennifer Salazar, MBS	USC
Yuliana Cabrera, BS	USC
Sarah Walter, MSc	USC
Lindsey Hergesheimen, BS	USC

San Francisco Veterans Affairs Medical Center

Thomas Neylan, MD	UC San Francisco
Jacqueline Hayes	UC San Francisco
Shannon Finley	UC San Francisco

Biostatistics Core Leaders and Key Personnel

Danielle Harvey, PhD	UC Davis (Core PI)
Michael Donohue, PhD	UC San Diego

MRI Core Leaders and Key Personnel

Clifford R. Jack, Jr., MD	Mayo Clinic, Rochester (Core PI)
Matthew Bernstein, PhD	Mayo Clinic, Rochester
Bret Borowski, RT	Mayo Clinic
Jeff Gunter, PhD	Mayo Clinic
Matt Senjem, MS	Mayo Clinic
Kejal Kantarci	Mayo Clinic
Chad Ward	Mayo Clinic

PET Core Leaders and Key Personnel

William Jagust, MD	UC Berkeley (Core PI)
Robert A. Koeppe, PhD	University of Michigan
Norm Foster, MD	University of Utah
Eric M. Reiman, MD	Banner Alzheimer's Institute
Kewei Chen, PhD	Banner Alzheimer's Institute
Susan Landau, PhD	UC Berkeley

Neuropathology Core Leaders

John C. Morris, MD	Washington University St. Louis
Nigel J. Cairns, PhD, FRCPath	Washington University St. Louis
Erin Householder, MS	Washington University St. Louis

Biomarkers Core Leaders and Key Personnel

Leslie M. Shaw, PhD	Perelman School of Medicine, UPenn
John Q. Trojanowki, MD, PhD	Perelman School of Medicine, UPenn
Virginia Lee, PhD, MBA	Perelman School of Medicine, UPenn
Magdalena Korecka, PhD	Perelman School of Medicine, UPenn
Michal Figurski, PhD	Perelman School of Medicine, UPenn

Informatics Core Leaders and Key Personnel

Arthur W. Toga, PhD	USC (Core PI)
Karen Crawford	USC
Scott Neu, PhD	USC

Genetics Core Leaders and Key Personnel

Andrew J. Saykin, PsyD	Indiana University
Tatiana M. Foroud, PhD	Indiana University
Steven Potkin, MD UC	UC Irvine
Li Shen, PhD	Indiana University
Kelley Faber, MS, CCRC	Indiana University
Sungeun Kim, PhD	Indiana University
Kwangsik Nho, PhD	Indiana University

Initial Concept Planning & Development

Michael W. Weiner, MD	UC San Francisco
Karl Friedl	Department of Defense (retired)

Part B: Investigators By Site

University of Southern California:
Lon S. Schneider, MD, MS
Sonia Pawluczyk, MD
Mauricio Becerra

University of California, San Diego:
James Brewer, MD, PhD
Helen Vanderswag, RN

Columbia University Medical Center:
Yaakov Stern, PhD
Lawrence S. Honig, MD, PhD
Karen L. Bell, MD

Rush University Medical Center:
Debra Fleischman, Ph.D.
Konstantinos Arfanakis, Ph.D.
Raj C. Shah, M.D.

Wien Center:
Dr. Ranjan Duara MD PI
Dr. Daniel Varon MD Co-PI
Maria T Greig HP Coordinator

Duke University Medical Center:
P. Murali Doraiswamy, MBBS
Jeffrey R. Petrella, MD
Olga James, MD

University of Rochester Medical Center:
Anton P. Porsteinsson, MD (director)
Bonnie Goldstein, MS, NP (coordinator)
Kimberly S. Martin, RN

University of California, Irvine:
Steven G. Potkin, MD
Adrian Preda, MD
Dana Nguyen, PhD

Medical University South Carolina:
Jacobo Mintzer, MD, MBA
Dino Massoglia, MD, PhD
Olga Brawman-Mintzer, MD

Premiere Research Inst (Palm Beach Neurology):
Carl Sadowsky, MD
Walter Martinez, MD
Teresa Villena, MD

University of California, San Francisco:
William Jagust MD
Susan Landau PhD
Howard Rosen, MD
David Perry

Georgetown University Medical Center:
Raymond Scott Turner, MD, PhD
Kelly Behan
Brigid Reynolds, NP

Brigham and Women's Hospital:
Reisa A. Sperling, MD
Keith A. Johnson, MD
Gad Marshall, MD

Banner Sun Health Research Institute:
Marwan N. Sabbagh, MD
Sandra A. Jacobson, MD

Sherye A. Sirrel, MS, CCRC

Howard University:
Thomas O. Obisesan, MD, MPH
Saba Wolday, MSc
Joanne Allard, PhD

University of Wisconsin:
Sterling C. Johnson, Ph.D.
J. Jay Fruehling, M.A.
Sandra Harding, M.S.

University of Washington:
Elaine R. Peskind, MD
Eric C. Petrie, MD, MS
Gail Li, MD, PhD

Stanford University:
Jerome A. Yesavage, MD
Joy L. Taylor, PhD
Ansgar J. Furst, PhD
Steven Chao, M.D.

Cornell University:
Norman Relkin, MD, PhD
Gloria Chiang, MD
Lisa Ravdin, PhD

ADNI Depression

Part A: Leadership and Infrastructure

Principal Investigator

Scott Mackin, PhD	University of California, San Francisco

ATRI PI and Director of Coordinating Center Clinical Core

Paul Aisen, MD	University of Southern California
Rema Raman, PhD	University of Southern California

Executive Committee

Scott Mackin, PhD	UC San Francisco
Michael Weiner, MD	UC San Francisco
Paul Aisen, MD	University of Southern California
Rema Raman, PhD	University of Southern California
Clifford R. Jack, Jr., MD	Mayo Clinic, Rochester
Susan Landau, PhD	UC Berkeley
Andrew J. Saykin, PsyD	Indiana University
Arthur W. Toga, PhD	University of Southern California
Charles DeCarli, MD	UC Davis
Robert A. Koeppe, PhD	University of Michigan

Data and Publication Committee (DPC)

Robert C. Green, MD, MPH	BWH/HMS (Chair)
Erin Drake, MA	BWM/HMS (Director)

Clinical Core Leaders

Michael Weiner MD	Core PI
Paul Aisen, MD	University of Southern California
Rema Raman, PhD	University of Southern California
Mike Donohue, PhD	University of Southern California

Clinical Informatics, Operations and Regulatory Affairs

Gustavo Jimenez, MBS	USC
Devon Gessert, BS	USC
Kelly Harless, BA	USC
Jennifer Salazar, MBS	USC
Yuliana Cabrera, BS	USC
Sarah Walter, MSc	USC
Lindsey Hergesheimer, BS	USC
Elizabeth Shaffer, BS	

Psychiatry Site Leaders and Key Personnel

Scott Mackin, PhD	UC San Francisco
Craig Nelson, MD	UC San Francisco
David Bickford, BA	UC San Francisco
Meryl Butters, PhD	University of Pittsburgh
Michelle Zmuda, MA	University of Pittsburgh

MRI Core Leaders and Key Personnel

Clifford R. Jack, Jr., MD	Mayo Clinic, Rochester (Core PI)
Matthew Bernstein, PhD	Mayo Clinic, Rochester
Bret Borowski, RT	Mayo Clinic, Rochester
Jeff Gunter, PhD	Mayo Clinic, Rochester
Matt Senjem, MS	Mayo Clinic, Rochester
Kejal Kantarci, MD	Mayo Clinic, Rochester
Chad Ward, BA	Mayo Clinic, Rochester
Denise Reyes, BS	Mayo Clinic, Rochester

PET Core Leaders and Key Personnel

Robert A. Koeppe, PhD	University of Michigan
Susan Landau, PhD	UC Berkeley

Informatics Core Leaders and Key Personnel

Arthur W. Toga, PhD	USC (Core PI)
Karen Crawford	USC
Scott Neu, PhD	USC

Genetics Core Leaders and Key Personnel

Andrew J. Saykin, PsyD	Indiana University
Tatiana M. Foroud, PhD	Indiana University
Kelley M. Faber, MS, CCRC	Indiana University
Kwangsik Nho, PhD	Indiana University
Kelly N. Nudelman	Indiana University

Part B: Investigators By Site

University of California, San Francisco:
Scott Mackin, PhD
Howard Rosen, MD
Craig Nelson, MD
David Bickford, BA
Yiu Ho Au, BA
Kelly Scherer, BS
Daniel Catalinotto, BA
Samuel Stark, BA
Elise Ong, BA
Dariella Fernandez, BA

University of Pittsburgh:
Meryl Butters, PhD
Michelle Zmuda, MA
Oscar L. Lopez, MD
MaryAnn Oakley, MA
Donna M. Simpson, CRNP, MPH
