# Supplementary material for: Mild-moderate CKD is not associated with cognitive impairment in older adults in the Alzheimer’s Disease Neuroimaging Initiative cohort
Source: PLoS One. 2020 Oct 9;15(10):e0239871. doi: 10.1371/journal.pone.0239871 (PMC7546911; doi:10.1371/journal.pone.0239871)
Supplement: Attachment — Submitted filename: Response to Reviewers (PONE-D-20-20489).docx [file pone.0239871.s003.docx]

**Response to Reviewers (PONE-D-20-20489)**

We thank the editors and the reviewers for their thoughtful feedback. We are also thankful for their suggestions which we believe have improved the manuscript. Our response to each concern is outlined below.

**Journal requirements**
One of the noted authors is a group or consortium Alzheimer’s Disease Neuroimaging Initiative. In addition to naming the author group, please list the individual authors and affiliations within this group in the acknowledgments section of your manuscript. Please also indicate clearly a lead author for this group along with a contact email address.

Response: ADNI has a large group of investigators mentioned in a 13 page PDF document for which the link is provided in the manuscript: <http://adni.loni.usc.edu/wp-content/uploads/how_to_apply/ADNI_Acknowledgement_List.pdf>. Since there are so many investigators, we presented the link rather than individual investigators. The ADNI recommends using this link too. Previous publications in Plosone (2 examples presented below) have also used this link rather than mentioning all the authors. 2 examples of Plosone publications for ADNI which have used this link are:

1. A ‘Comprehensive Visual Rating Scale’ for predicting progression to dementia in patients with mild cognitive impairment Jang, Jae-Won, Park, Jeong Hoon, Kim, Seongheon, Park, Young Ho, Pyun, Jung-Min, Lim, Jae-Sung, Kim, Youngho, Youn, Young Chul, Kim, Sang Yun and Initiative, Alzheimer’s Disease Neuroimaging

PMID: 30125290; 2018; Journal PloS one; vol. 13; no. 8; pp. e0201852; AW5HwZDTMOwVf_qbJr_0

2. Prediction and classification of Alzheimer disease based on quantification of MRI deformation

X. Long, L. Chen, C. Jiang, L. Zhang and I. Alzheimer's Disease Neuroimaging PMCID:5338815; 2017; Journal PLoS One; vol. 12; no. 3; pp. e0173372; AWUWwDjFdzjauPDbjhtH doi:10.1371/journal.pone.0173372

If the journal still requires us to name the investigators, we can.

**Editors:**

1) Normal vs declined eGFR should be analyzed.

Response: We have added another multivariable linear regression analysis with this stratification of eGFR (Supplementary Table 1).

2) You claimed this article is focus on aged cohort but the cohort selection is not clearly described in regards of age.

Response: We agree. We have clarified the age under methods and described the cohort in more detail under introduction in the revised manuscript.

**Reviewer #1**

1) It is considered that eGFR correlates with the measured GFR in patients with renal dysfunction, while it is doubtful that the same correlation is shown in patients with normal renal function. How would you describe that point?

Response: We agree and have clarified this further in our limitations. Obtaining measured GFR is cumbersome and impractical, eGFR is often used in both research and clinical practice. Since our study is essentially a null study and there is no association between any level of eGFR and cognition (Figure 1, Table 2), this does not alter our results or conclusion.

2) BMI should be entered into all multivariable models.

Response: We have added BMI in the multivariate models.

3) I wonder whether the decorrelation between eGFR and cognition in the multivariable linear regression analysis would be maintained when stratified by eGFR (eGFR<60 or eGFR ≥60). The authors should show the results stratified by eGFR.

Response: We have added this stratification of eGFR (Supplementary Table 1).

4) The authors describe “Unadjusted correlation analysis showed weak negative associations of ADNI-Mem scores and ADNI-EF scores with eGFR with correlation coefficients of -0.105 (p=0.001) and -0.02, p=0.43) respectively (Figure 1)” in Page 8 and “Weak correlation between eGFR and ADNI-Mem and ADNI-EF scores were again seen (Supplementary Figure 2)” in Page 10 in the Results section. These descriptions are incorrect because some of these associations are not significant. The authors should revise these descriptions.

Response: Thank you for pointing this out. We have revised the descriptions as suggested.

**Reviewer #2:**

1) Could the authors include more details on entry criteria for the parent study, I don't see age criteria mentioned, perhaps include age range to clarify whether the cohort was restricted to older adults or not?

Response: We have added the mean age (74) in ADNI in the introduction and the age range (55-90) in the methods section where we describe the ADNI cohort. For ADNI 2 the minimum age for the normal control group and the significant memory concern group was 65 years.

2) Much of the rationale for why the authors chose to conduct this study in this particular cohort does not come out until the discussion. I believe the argument is that this is an older cohort without much underlying comorbidity other than dementia or mild cognitive impairment for some? Presenting this in the introduction could provide a stronger rationale for why the authors chose to conduct this study in this particular cohort.

Response: We agree. We have added this detail in the last paragraph of the introduction in the revised manuscript.

3) In the introduction, it would be good to present more detail on the demographic characteristics of study populations in which the association between eGFR and cognitive function has been measured. The introduction makes it sound like there have been no prior studies of cognitive impairment and eGFR in older populations? My sense is that some prior studies have been conducted in older adults, so summarizing those studies and their results would be helpful then highlighting original contribution of this study (it sounds like the contribution of this study is that it included mostly healthy older adults?)

Response: We agree and have added more detail about other published studies as suggested.

4) Consider less sweeping title, the authors found no association of eGFR in this particular cohort, but I am not sure this justifies the statement that there is no association in "the elderly." Maybe no association in older adults enrolled in the Alzheimer’s Disease Neuroimaging initiative?

Response: Thank you for this suggestion. We have changed the title to ‘Mild-moderate CKD is not associated with cognitive impairment in older adults in the Alzheimer’s Disease Neuroimaging Initiative cohort’ to more accurately describe this study.
